# Supplementary figures and images for: Effects of Biotin on survival, ensheathment, and ATP production by oligodendrocyte lineage cells in vitro
Source: PLoS One. 2020 May 29;15(5):e0233859. doi: 10.1371/journal.pone.0233859 (PMC7259710; doi:10.1371/journal.pone.0233859)

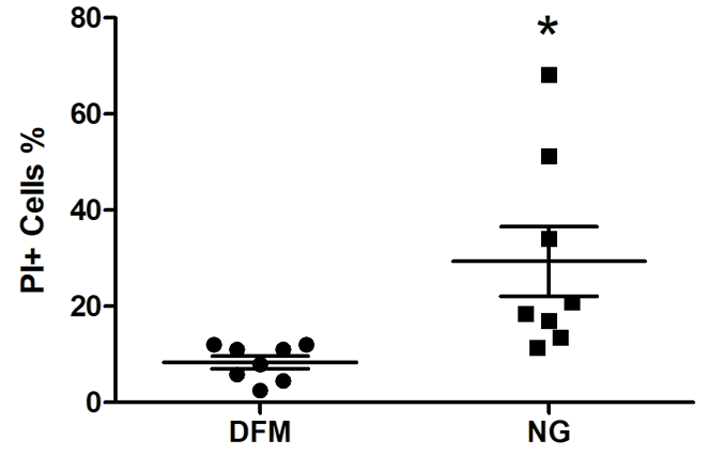

Supplement: S1 Fig — OPCs were cultured in DFM control or NG conditions for 24 hrs. OPCs were immunostained with monoclonal antibody O4 and PI. PI+ cells were analyzed using a MATLAB program described in the methods. Comparison between DFM and NG was performed by paired t-test, p = 0.02, N = 8. (TIF) [file pone.0233859.s001.tif]

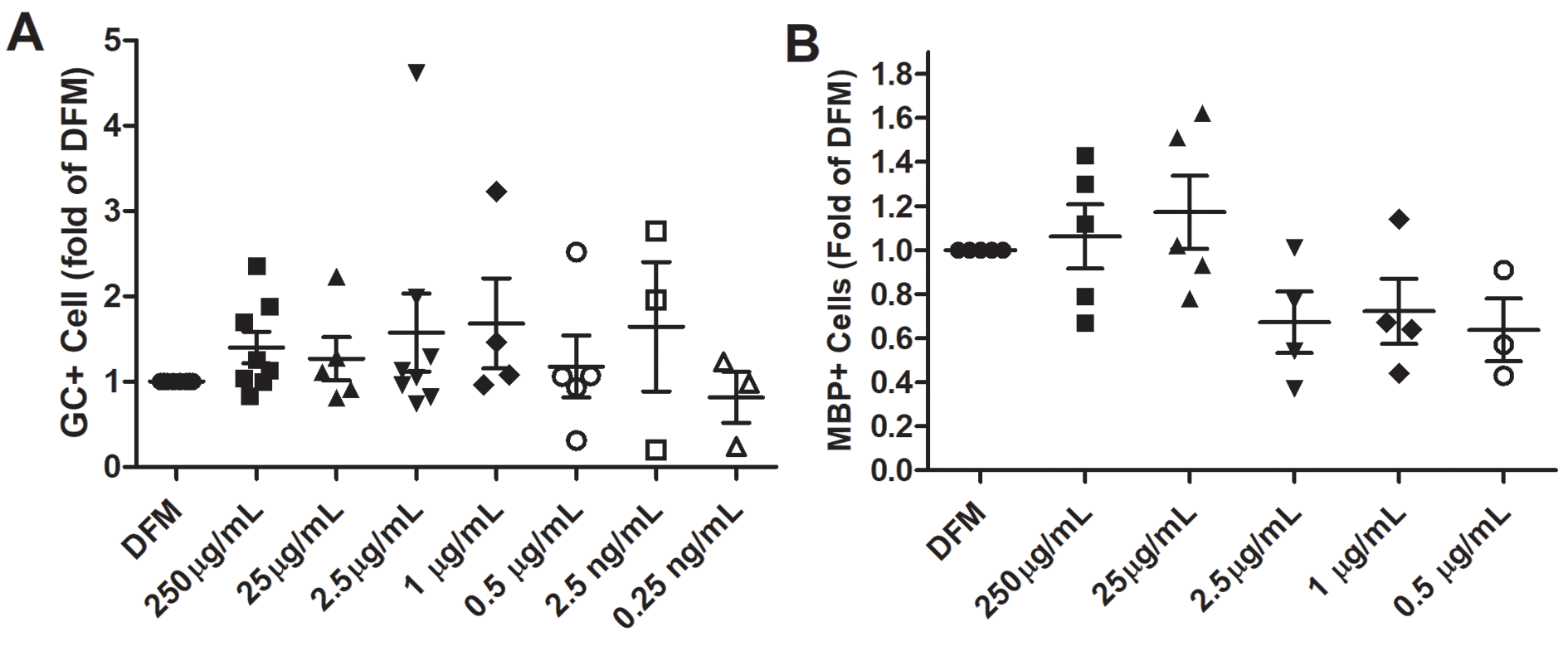

Supplement: S2 Fig — Panel A, OPCs were treated with indicated concentrations of biotin in DFM for 24 hrs. OPCs were immunostained with monoclonal antibodies O4 and GC. Cells were imaged and GC+ cells were analyzed using a MATLAB program described in the methods. Panel B, OPCs were treated with indicated concentrations of biotin in DFM for 3 days. OPCs were immunostained with monoclonal antibodies O4 and MBP. 1-way ANOVA was performed followed by Dunnett’s multiple comparison test. (TIF) [file pone.0233859.s002.tif]

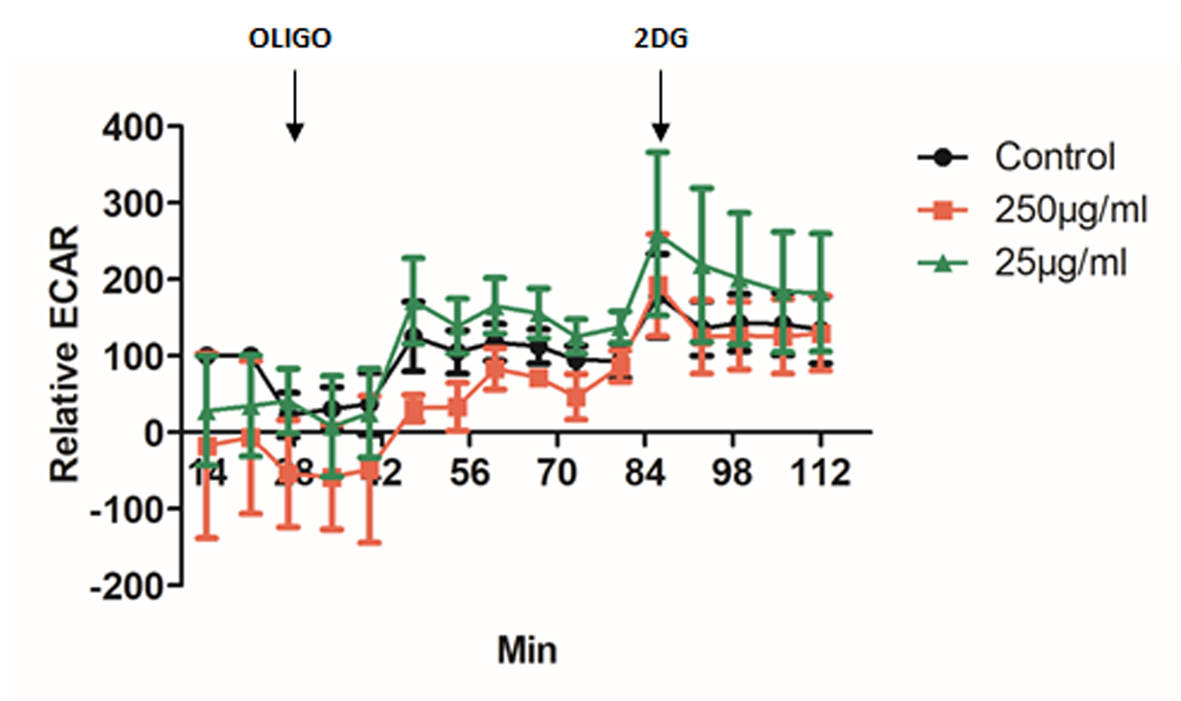

Supplement: S3 Fig — ECAR data normalized to basal values under control conditions that ranged from 0.03 to 0.3 pmol O2/min/μg protein. The time point for applying OLIGO and 2DG is indicated. (TIF) [file pone.0233859.s003.tif]
