# Supplementary material for: Effects of Biotin on survival, ensheathment, and ATP production by oligodendrocyte lineage cells in vitro
Source: PLoS One. 2020 May 29;15(5):e0233859. doi: 10.1371/journal.pone.0233859 (PMC7259710; doi:10.1371/journal.pone.0233859)
Supplement: Attachment — Submitted filename: Review.docx [file pone.0233859.s004.docx]

Reviewer remarks on the article: “Effects of Biotin on survival, ensheathment, and ATP production by oligodendrocyte lineage cells in vitro “.

In the context of multiple sclerosis, the authors study the influence of a high dose of biotin on oligodendrocyte precursor cells (OPCs) under metabolic stress (low-glucose concentration in the medium). The OPC cell death is increased in this condition, but the increase can be reduced in presence of a high concentration of biotin. The authors also used nanofiber myelination assays to show that biotin increases the percentage of ensheathing cells, the number of ensheathed segments per cell, and the length of ensheathed segments. They also show that in cell culture, biotin increases oxygen consumption rate and ATP production.

The article is well written and interesting.

Here are my remarks:

1- There are several names that should be explained, so that non-specialists could fully understand the paper:

- A2B5

- what are mitotoxins and what are their effects?

- same question with oligomycin.

- In the Materials and Methods section, please give the concentration of antibodies in the Proliferation and Protection assays and the Nanofiber ensheathment assay paragraphs, so that the experiments could be reproduced.

2. Figures

In Figure 1 and 2, the conditions significantly different from the control do not apple clearly. For Example, in Figure 1A: it seems that only the concentrations of 250 and 2.5 ug/mL are significantly different from the control (the only two points with an asterisk). However, at the beginning of the Result Section, it is written: "As shown in Figure 1A, supplementation with biotin over a concentration range of 2.5 to 250 μg/ml significantly reduced the % PI+ cells (mean % reduction 35+-5 %, n = 8, p < 0.001 at 250 μg/ml).

An asterisk should be put above all the data points, or the sentence should be changed.

- Same remark for Figure 2A: is only the 250 ug/mL condition statistically significant? If this is the case, it should be mentioned in the text.

- Figure 2C: I do not see the experimental data points, it seems that it is only a fit that is showed here. Please add the experimental data points, so that I could see the error bars. How are estimated the error bars on the proportion of length of sheath segment per cell? are the differences statistically significant?

- Figure 2 D and E: the figures of cells on nanofibers are not very clear, the contrast is not the same and it is really difficult to see a difference. Would it be possible to show a magnified image so that we could clearly see a few ensheathed nanofibers?

3. Metabolism

In Rao et al, PlosOne 2017 (from the same group), it is said that "adult rat oligodendrocytes preferentially use glycolysis whereas [..] oligodendrocyte progenitor cells from which they are derived, mainly use oxidative phosphorylation to produce ATP". In this article, OPCs from the brains of newborn Sprague-Dawley rats were used, as well as human adult brain derived OLs. These two types of cells should have a different metabolism. How does metabolism (glycolysis or oxidative phosphorylation) influence the effect of biotin?
